# Accelerated age-related olfactory decline among type 1 Usher patients

**DOI:** 10.1038/srep28309

**Published:** 2016-06-22

**Authors:** João Carlos Ribeiro, Bárbara Oliveiros, Paulo Pereira, Natália António, Thomas Hummel, António Paiva, Eduardo D. Silva

**Affiliations:** 1 Department of Otorhinolaryngology, Coimbra University Hospitals, Portugal; 2 Faculty of Medicine, University of Coimbra, Portugal; 3 Centre of Ophthalmology and Vision Sciences, IBILI, Faculty of Medicine, University of Coimbra, Portugal; 4 Department of Otorhinolaryngology, TU Dresden, Dresden, Germany

## Abstract

Usher Syndrome (USH) is a rare disease with hearing loss, retinitis pigmentosa and, sometimes, vestibular dysfunction. A phenotype heterogeneity is reported. Recent evidence indicates that USH is likely to belong to an emerging class of sensory ciliopathies. Olfaction has recently been implicated in ciliopathies, but the scarce literature about olfaction in USH show conflicting results. We aim to evaluate olfactory impairment as a possible clinical manifestation of USH. Prospective clinical study that included 65 patients with USH and 65 normal age-gender-smoking-habits pair matched subjects. A cross culturally validated version of the Sniffin’ Sticks olfaction test was used. Young patients with USH have significantly better olfactory scores than healthy controls. We observe that USH type 1 have a faster ageing olfactory decrease than what happens in healthy subjects, leading to significantly lower olfactory scores in older USH1 patients. Moreover, USH type 1 patients showed significantly higher olfactory scores than USH type 2, what can help distinguishing them. Olfaction represents an attractive tool for USH type classification and pre diagnostic screening due to the low cost and non-invasive nature of the testing. Olfactory dysfunction should be considered among the spectrum of clinical manifestations of Usher syndrome.

Usher syndrome (USH) is a heterogeneous, rare, multisystemic genetic disease, characterized by sensorineural hearing loss, retinitis pigmentosa (RP), and variable vestibular dysfunction. Its prevalence ranges from 3 to 8.0 per 100,000 people^1^.

Patients with Usher syndrome (USH) often display a complex array of signs and symptoms. The visual, auditory, and vestibular manifestations are well documented, while additional manifestations of the condition have not received much attention. USH is classified into three clinical types^2^. USH type 1 (USH1) is associated with severe-to-profound sensorineural congenital deafness, vestibular areflexia, and prepuberal onset of RP. USH type 2 (USH2) is characterized by moderate-to-severe hearing impairment, no vestibular impairment, and onset of RP in the first or second decade of life. In type 3 (USH3), the least prevalent type of USH, the post lingual hearing loss onset is progressive while RP and vestibular dysfunction onset is variable^3^.

While auditory and vestibular testing helps to differentiate among USH types, overlapping and atypical presentations have been described for all three types of Usher syndrome^4^,^5^,^6^,^7^,^8^. Specific genetic patterns are associated with each USH phenotype, even while genetic heterogeneity has been reported^9^. A genotype-phenotype correlation would help to guide genetic evaluation or even make it unnecessary if a perfect correlation is achieved. Next generation sequencing provides a useful tool to reveal clinical misdiagnosis, identify late onset syndromic features, establish novel genotype/phenotype correlations, and to facilitate clinical diagnosis^10^,^11^. Yet, our group has observed 10% of cases manifesting atypical presentations (unpublished data).

Improving phenotype may help improving USH diagnosis and genotype-phenotype correlation. Olfaction may be an important link in USH. Nevertheless, very few papers studied olfaction in patients with USH. Seeliger *et al*.^12^ studied 8 USH1 and 31 USH2 patients with *Sniffin’ Sticks* test^13^. Although no significant olfactory differences were found, it is interesting to observe that patients with USH1 were 20–40 years old and showed a median olfactory score higher than control group (9.7 vs 8.5). They also found a far more rapid decline in olfactory threshold of USH1 that was not explained by normal ageing. Zrada *et al*.^14^ observed 22 patients (8 USH1 and 22 USH2), by means of UPSIT age- gender- smoking-habit- matched controls^15^. Olfactory thresholds were higher than those of controls, although statistical significance was not attained, probably because the study was not adequately powered. In another study, Marietta *et al*.^16^ studied the olfactory acuity in a subgroup of USH1C patients. They used UPSIT test in 18 older patients, not pair matched. Although an age decline in olfaction appeared to be greater than normative data, the difference was not significant^16^.

Patients with ciliopathies affecting the inner ear are frequently deaf and/or persons who exhibit balance difficulties; and patients with retinal ciliopathies often become blind^17^. Genetics, biochemistry and proteomic approaches have demonstrated that USH proteins are organized in networks in both the eye and the inner ear^18^. Consequently, a disease that have both systems affected like USH is expectable to be a sensory ciliopathy^18^. Furthermore, USH has been associated with bronchiectasis, chronic sinusitis, reduced nasal mucociliary clearance^19^,^20^ and altered sperm function^21^, all of which are suggestive of ciliary dyskinesia^19^.

In recent years a number of papers helped to elucidate the relationship between olfaction and its genetic underpinnings since the pioneering work of Buck and Axel, 1991^22^. Because recent evidence implicated olfaction in sensory ciliopathies^18^,^23^,^24^,^25^,^26^,^27^,^28^, it is expectable that olfaction is also implicated in USH, however, the few previous studies addressing functional olfactory acuity in patients with USH have showed conflicting results^12^,^14^,^29^.

## AIM

Knowing that Usher syndrome is a ciliopathy from a histopathological and molecular standpoint, we have undertaken a prospective recruitment of patients with USH to study their functional olfactory features. This study aimed at identifying and characterizing putative differences in olfactory capacity between patients with USH and controls, as well as among the subtypes of USH.

## Patients and Methods

All participants provided informed written consent. The study followed the Declaration of Helsinki 2013 on Biomedical Research Involving Human Participants and was approved by the Ethics Committee of the Faculty of Medicine, University of Coimbra, Portugal.

Sixty-five patients with USH (49.2 ± 15.1 years old; 18 females and 47 males) were prospectively recruited from the Otorhinolaryngology consultation of the Centro Hospitalar e Universitário de Coimbra, Portugal. They were pair-matched with 65 healthy controls (controls 49.2 ± 14.9 years old) for age, sex, and smoking habits, so that a total of 130 participants were studied.

All participants received a full ENT clinical examination, as well as a structured interview, a rhinologic examination including nasal endoscopy and a standardized *Sniffin*’ *Sticks* test (Burghart GmbH, Wedel, Germany) culturally validated to the Portuguese population (SnSt-pt)^30^. Briefly, this test comprised three subtests, namely odor threshold (T, tested by means of a single staircase procedure), odor discrimination (D, 3-alternative forced choice) and odor identification (I, 4-alternative forced choice). Results of the 3 subtests are typically summed up and presented as a composite TDI score, as review by Hummel *et al*.^31^.

Eligible patients had a documented neurosensory hearing loss and RP, fulfilling the clinical criteria for USH1 or USH2, as defined by the USH consortium^2^. Exclusion criteria included participants with known factors affecting olfaction, such as post-traumatic olfactory dysfunction, sinonasal disease, malignant tumor, recent radiotherapy or chemotherapy, post-upper respiratory tract infection, use medication known to interfere in olfaction^32^, and Parkinson´s and Alzheimer´s disease.

Statistical analysis of the data was performed using the *Statistical Package for the Social Sciences* (SPSS), version 22.0 (SPSS, Inc., Chicago, IL). The normality of continuous variables was tested with the *Kolmogorov-Smirnov* test. Age is displayed as mean and standard deviation. Olfactory data is presented as median and interquartile range. The *Mann-Whitney* U test or *Student*’*s* t-test test was used whichever appropriate. In spite of the main measure being the *Sniffin*’ *Sticks* TDI scores, and though it would be justifiable not to perform multiple comparisons adjustment in planned comparisons using each one of the partial measures (T, D, and I sub-scales of the Sniffin’ Sticks TDI scale), the fact is that those sub-scales are not perfectly independent. As a result, the multiple comparisons problem arises, and is sorted out through the *Holm*’*s* correction, which controls the Family-Wise Error Rate, and the *Benjamini-Hosper* correction, which controls the False Discovery Rates. *Spearman´s* rank correlation coefficient was calculated to verify the relationship between subgroups tested for olfaction. Multiple linear regression with previous bootstrapping was used to correlate olfaction in relation to age and Usher subtype. All tests were two-tailed and statistical significance was accepted at the p < 0.05 level.

## Results

Sixty-five USH patients (49.2 ± 15.1 years old; 18 females/47 males) were pair-matched with 65 healthy controls (49.2 ± 14.9 years old; 18 f/47 m).

Patients with USH included 22 USH1 (43.4 ± 16.7 [18–77] years old; 5 f/17 m) that were compared to 22 healthy pair matched controls (43.3 ± 16.1 years old; 5 f/17 m), and another 43 USH2 (52.2 ± 13.5 years old; 13 f/30 m) that were pair matched to 43 healthy controls (52.2 ± 13.5 [32–78] years old; 13 f/30 m).

### Total USH patients’ vs controls

USH patients′ olfactory scores were compared to pair matched controls. Patients with USH had significantly better SnSt- composite TDI olfaction scores than healthy controls (32.8 [28.5–37.4]) vs (30.0 [28.6–31.3]), (p = 0.002). Both USH1 and USH2 showed better discrimination scores than controls, with large standardized effect sizes (Table 1).

### USH1 VS USH2

USH1 and USH2 are distinguishable in terms of hearing loss and vestibular function. Patients with USH1 and USH2 were compared regarding olfaction capabilities and USH1 showed significantly higher TDI and identification scores than USH2 (Table 2). Effect size was 1.27 with *a posteriori* power of 99.87%.

### Usher syndrome olfactory age decline

A deeper look showed a significant olfactory ageing decline in patients with USH. Because olfaction is known to decline with ageing^30^ and to adequately evaluate how olfactory ability is affected in USH patients, it is important to rule out ageing´s effect on olfaction. Consequently, after considering the normal age effect on olfaction based on our normative data^30^ (n = 203, that compares well with original data from Hummel *et al*.^31^), a multiple regression model was obtained. Patients with USH show a significantly faster age decline in olfaction. This effect is particularly relevant regarding USH1 patients: *TDI* = 50,010 − 0,401 × *age*; *T* = 16,549 − 0,188 × *age*; *D* = 16,248 − 0,124 × *age*; and *I* = 17,096 − 0,127 × *age* (Table 3).

After correcting for normal olfactory ageing decline, patients with USH1 show a significantly faster olfactory decline with age than healthy subjects with a R^2^ = 0.703 and a power of 99.99% (Table 4).

**Figure 1:**
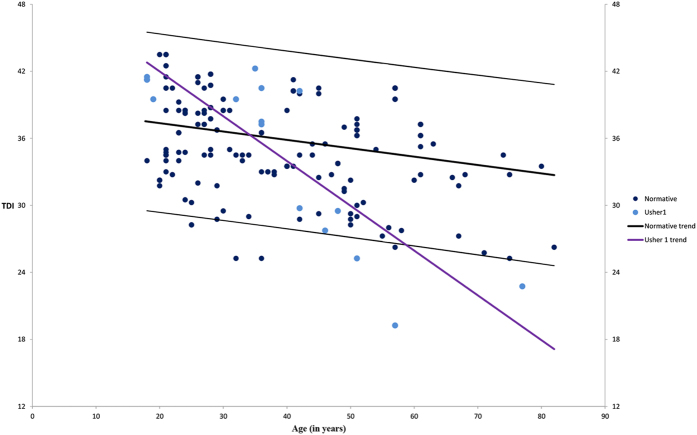
USH1 faster olfactory ageing decline, not expected by normal age olfaction decline (p < 0.001). USH1: Usher type 1. TDI: composite score of Sniffin’ Sticks olfaction test.

The significant olfactory decline in patients with USH1, not attributable to the olfactory ageing effect, is displayed in Fig. 1. Normative data is presented for comparison purposes (Fig. 1).

**Figure 2:**
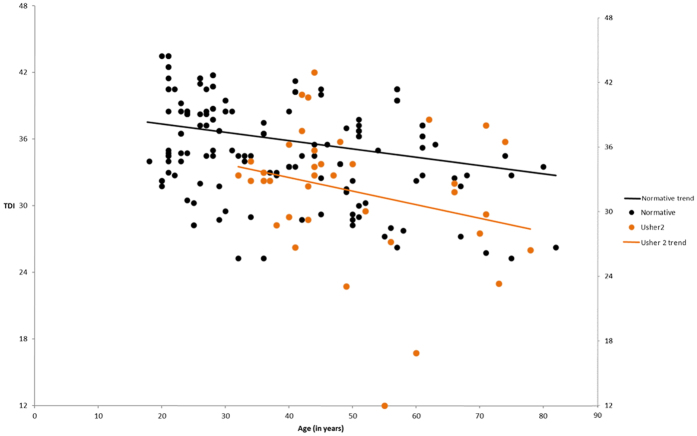
USH2 olfactory ageing decline comparing to the healthy population (p > 0.05). USH2: Usher type 2. TDI: composite score of Sniffin’ Sticks olfaction test.

Considering patients with USH2, none of the models were statistically significant. Olfactory ageing effect is presented in Fig. 2.

When comparing patients with USH1 younger or older than the mean age of the group (43 years old), the older ones show significantly lower TDI scores (11.9 ± 1.7 vs 4.9 ± 2.0, p < 0.001) (Table 5). Such effect is not seen in patients with USH2.

The faster olfactory loss after adjusting for normal age related olfactory loss comparisons achieved a power of at least 91.12% in the all comparisons.

## Discussion

The present study shows that patients with USH have significantly better TDI scores than healthy controls. Moreover, patients with USH1 show significantly higher TDI scores than patients with USH2, what can help to phenotypically distinguish them, having important clinical implications. A cut-off age of 43 years could be used. We observe that patients with USH1 have a faster decrease in olfactory function than what happens in healthy subjects, leading to much lower olfactory scores in older patients with USH1.

Data presented in this study indicates that olfactory tests may provide a valuable tool in characterizing and categorizing USH types. Although auditory and vestibular testing helps to distinguish typical USH patients, additional phenotypic abnormalities are necessary to aid in differentiating among atypical cases. Because of that, the current phenotype–genotype correlation is insufficient to predict the likely causative mutation, which makes sequencing of all known USH genes an often necessary but difficult way to identify the underlying genetic defect in affected patients.

The few papers addressing olfaction in Usher have shown conflicting results^12^,^14^,^16^,^29^. Our study tried to solve some methodology problems that highly limited the strength of conclusions from those papers. Being a rare disease, with such heterogeneous phenotypes, it is difficult to investigate a sample size with a sufficient power to confirm possible differences in olfaction among patients with Usher^12^,^14^,^16^. While some papers trust on the subjects’ self-assessment of olfaction, it is unreliable, and testing olfactory function is necessary^33^. Moreover, some previous studies used non-standardized and not validated olfactory testing^14^. Additionally, because patients were not matched for well-known factors to affect olfaction^13^,^34^,^35^,^36^ including age, gender and smoking habits^12^,^14^,^37^, those studies must be interpreted with caution^38^.

The better olfaction observed in young USH1 may be easily explained by the adaptive intramodal neuroplasticity and cross-modal sensory reorganization associated with blindness and by the behavioral compensations taking place in the remaining senses, including those in the tactile, auditory and olfactory domains^39^,^40^,^41^,^42^. Recent studies on olfaction in blind persons have shown that early blindness may affect olfactory processing^39^,^41^,^43^,^44^,^45^. Not all studies have found superior olfactory capabilities in the different olfaction areas^46^,^47^,^48^, but discrepancies are not surprising because most studies enrolled few patients, lacked matched-pair controls and did not use standardized and cultural validated olfactory testing procedures^46^,^47^,^49^,^50^. Patients with USH2 may have shown less significant differences because when a person is afflicted by combined visual and hearing impairment, it becomes more difficult for other sensory systems to compensate^39^.

The higher scores observed in younger patients with USH, in particular in USH1, may be a compensatory olfactory effect of deaf-blindness. There is growing evidence that congenitally blind individuals outperform age- and gender matched, normally sighted persons in tactile, auditory and possibly also olfactory tasks^39^,^41^,^44^, but such effect is not seen in late-blindness patients^41^,^45^,^51^. Thus, our results are not surprising in view of the fact that both hearing and visual loss occur earlier and in a more severe manner in patients with USH1^44^.

The number of cases used to perform each regression was different due to the sampling of such a rare disease, where patients with USH1 were 22 and USH2 43. In spite of the different number of cases used in both regressions, statistical power was similar thus both regressions are valid and comparable in terms of conclusions achieved. The regression models showed a power superior to 91% in all comparisons performed. When the observed effect sizes are large (between 1.27 and 4.24), a nearly null type II error exists and the p-value achieved (p < 0.001) represent a true positive value between groups. The difference between patients with USH1 and USH2 concerning TDI scores represent an effect size of 1.27 which means that the achieved *a posteriori* power with a sample of 22 and 43 patients is 99.87%. Therefore, we must conclude there is no bias related to the different number of patients in each group and consequently, the observed dispersion is related to the olfactory features of patients with USH.

Data linking USH to a ciliopathy is grounded on histopathological and molecular studies^24^,^27^. Most of the USH proteins have been localized at or around ciliary photoreceptor structures in the retina (as well as other ciliated tissues, such as olfactory epithelium)^14^,^21^,^52^,^53^. On the other hand, a considerable body of evidence indicates that olfactory dysfunction might well be associated with ciliary disorders^18^,^25^,^27^,^53^,^54^. Proteins related to the USH have a periciliary localization and play a role in ciliogenesis and ciliary maintenance of olfactory cilia^55^. Furthermore, given the evidence linking ciliary abnormalities to USH- related pathology in the retina^18^,^56^, inner ear^18^,^57^, nasal^27^,^52^,^58^ and tracheal mucosa^19^,^59^,^60^, and sperm^21^, the consequences of a genetic olfactory cilia impairment^21^,^24^,^52^,^56^ may well explain the faster decrease in olfaction seen in this study´s patients with USH1.

Although it is the biggest study on olfaction in patients with USH to our knowledge, the results must be regarded with caution until larger series confirm it. Consequently, and because of the rarity of USH, multicenter studies are needed to investigate olfactory function in these rare cases. Moreover, a possible supra-threshold effect may have not been studied due to the possible ceiling effect of *Sniffin*’ *Sticks*, something that was not explored^41^.

Olfactory dysfunction should be considered in the spectrum of clinical manifestations associated with Usher syndrome. Patients with USH present better olfactory scores than controls, especially younger USH1. The olfaction difference between USH1 and USH2, as well as the major decline in olfactory ability seen in USH1, but not in USH2, may contribute to distinguish USH types and represent an attractive tool for pre diagnostic screening due to the low cost and non-invasive nature of the procedure.

## Additional Information

**How to cite this article**: Ribeiro, J. C. *et al*. Accelerated age-related olfactory decline among type 1 Usher patients. *Sci. Rep.* **6**, 28309; doi: 10.1038/srep28309 (2016).

## Figures and Tables

**Table 1:** Sniffin’ Sticks Portuguese version olfactory test results comparing Usher and Usher type patient’s vs controls.

		Patients	Controls	STS	p	Holm’s Correction (α)	Benjamini-Hosper (α)
***USH***	T	8.3 (5.1–11.0)	7.8 (5.8–8.3)	−1.585	0.113	0.025	0.033
	D	11 (9–12)	10 (9–11)	**−3.416**	**0.001**	**0.017**	**0.017**
	I	13 (11–14)	13 (12–13)	−0.476	0.634	0.050	0.050
	TDI	32.8 (28.5–37.4)	30.0 (28.6–31.3)	**−3.095**	**0.002**	–	–
***USH1***	T	8.1 (4.6–12.5)	7.9 (5.8–9.4)	−0.646	0.518	0.050	0.050
	D	12.5 (9.3–13.8)	9.5 (9–11)	**−2.212**	**0.027**	**0.017**	**0.017**
	I	14 (11.3–15)	13 (12–13)	−1.104	0.270	0.025	0.033
	TDI	38.5 (28.2–41.1)	30.9 (29.1–32.3)	−1.623	0.105	–	–
***USH2***	T	8.3 (5.5–10.8)	7.5 (5.5–8.3)	−1.794	0.073	0.025	0.033
	D	11 (9–12)	10 (9–11)	**−2.613**	**0.009**	**0.017**	**0.017**
	I	12 (11–14)	13 (12–13)	0.072	0.943	0.050	0.050
	TDI	32.3 (28.8–35)	29.8 (27.5–30.5)	**−2.856**	**0.004**	**–**	–

STS: Standardized Test Statistic. TDI: composite score of T, D and I. T: threshold; D: discrimination; I: Identification. Highlighted differences should be retained as statistically significant differences, according to the alpha level presented either by the Holm’s or the Benjamini-Hochberg correction for multiple comparisons.

**Table 2:** Sniffin’ Sticks Portuguese version olfactory test results comparing olfaction between USH1 with USH2.

	USH1	USH2	STS	p	Holm’s Correction (α)	Benjamini-Hochberg (α)
**T**	8.1 (4.6–12.5)	8.3 (5.5–10.8)	**−4.020**	**<0.001**	**0.017**	**0.017**
**D**	12.5 (9.3–13.8)	11 (9–12)	0.093	0.926	0.050	0.050
**I**	14 (11.3–15)	12 (11–14)	**−3.360**	**0.001**	**0.025**	**0.033**
**TDI**	38.5 (28.2–41.1)	32.3 (28.8–35)	**−4.261**	**<0.001**	–	–

STS: standardized test statistic. TDI: composite score of T, D and I. T: threshold test; D: discrimination test; I: Identification test. Highlighted differences should be retained as statistically significant differences, according to the alpha level presented either by the Holm’s or the Benjamini-Hochberg correction for multiple comparisons.

**Table 3:** Linear regression values show the ageing olfactory loss in USH1 patients, compared with normative data.

	Correlation		Regression		
	Coefficient	P	Coefficient	95% confidence interval	p
**T**	−0.792	<0.001	**−0.188**	−0.268 a −0.134	0.001
**D**	−0.824	<0.001	**−0.124**	−0.192 a −0.093	0.002
**I**	−0.842	<0.001	**−0.127**	−0.175 a −0.082	0.001
**TDI**	−0.872	<0.001	**−0.401**	−0.575 a −0.295	0.002

TDI = composite score of T, D and I olfactory tests. T = threshold test; D = discrimination test; I = Identification test. All the p-values remained statistically significant either by the Holm’s or the Benjamini-Hosper correction for multiple comparisons.

**Table 4:** Decreased olfactory function related to USH1 after adjusting for age related olfactory loss.

	Healthy subjects age dependent olfactory decrease			Usher Syndrome age dependent olfactory decrease		
	Mean	95% CI	p	Mean	95% CI	P
T	−0.032	−0.058 to −0.006	0.017	**−0.156**	−0.225 to −0,087	<0.001
D	−0.018	−0.037 to +0.001	0.067	−**0.106**	−0.157 to −0.056	0.001
I	−0.025	−0.040 to −0.010	0.001	**−0.102**	−0.142 to −0.062	<0.001
TDI	−0.075	−0.112 to −0.038	<0.001	**−0.326**	−0.476 to −0.177	<0.001

TDI: composite score of T, D and I olfactory tests. T: threshold test; D: discrimination test; I: Identification test.; CI – confidence interval. Highlighted differences should be retained as statistically significant differences.

**Table 5:** Olfaction scores comparing USH, USH1 and USH2 patients below and above 43 years old (mean age of USH1), after correcting for normal ageing decline.

		Age		STS	P	Holm’s Correction (α)	Benjamini-Hochberg (α)
		<43yr	≥43yr				
***USH***	T	10.4 ± 2.3	6.8 ± 3.2	**−4.020**	**<0.001**	**0.017**	**0.017**
	D	11.8 ± 1.9	10.1 ± 2.0	0.093	0.926	0.050	0.050
	I	13.6 ± 1.5	11.6 ± 2.6	**−3.360**	**0.001**	**0.025**	**0.033**
	TDI	35.8 ± 4.8	29.7 ± 6.8	−4.261	<0.001		
***USH1***	T	11.9 ± 1.7	4.9 ± 2.0	**−3.915**	**<0.001**	**0.017**	**0.017**
	D	12.9 ± 1.2	8.4 ± 1.1	**−3.112**	**<0.001**	**0.050**	**0.050**
	I	14.4 ± 1.0	10.2 ± 1.6	**−3.201**	**<0.001**	**0.025**	**0.033**
	TDI	39.1 ± 3.5	24.9 ± 4.1	**−3.120**	**<0.001**		
***USH2***	T	9 ± 1.9	7.5 ± 3.3	0.223	0.232	0.017	0.017
	D	10.8 ± 1.9	10.4 ± 1.9	0.466	0.491	0.050	0.050
	I	12.8 ± 1.6	11.9 ± 2.7	0.409	0.432	0.025	0.033
	TDI	32.7 ± 3.8	30.7 ± 6.9	0.516	0.532		

USH: Usher syndrome patients. USH1: Usher type 1 patients. USH2: Usher type 2 patients. TDI: composite score of T, D and I olfaction tests. T: threshold test; D: discrimination test; I: Identification test. Highlighted differences should be retained as statistically significant differences, according to the alpha level presented either by the Holm’s or the Benjamini-Hochberg correction for multiple comparisons.
